# Understanding the Predictive Factors that Influence the Perceived Burden of Dementia Caregivers

**DOI:** 10.1093/geroni/igab046.3545

**Published:** 2021-12-17

**Authors:** Kaitlyn Lucas, Katherine Judge

**Affiliations:** 1 Cleveland State University, Westlake, Ohio, United States; 2 Cleveland State University, Cleveland, Ohio, United States

## Abstract

Caregivers of individuals with dementia have increased levels of burden because of their caregiving duties which in turn results in negative effects on their physical, emotional, and mental wellbeing. Few studies have examined individual difference-based constructs such as positive and negative affect in relation to caregiver burden. Positive affect is characterized by pleasant emotions and expressions and higher levels of overall life satisfaction whereas negative affect is characterized by more pessimistic emotions and expressions and lower overall life satisfaction. Analyses were performed on dementia caregivers (n=102) using the online data collection tool MTurk Prime. The average caregiver provided 21 or more hours of care with 76.9% of caregivers living with their loved one with dementia. Significant correlations were found between caregiving burden and positive affect (r= -.31, p=.002) and negative affect (r= .56, p=<.001), with higher positive affect related to lower burden and higher negative affect related to greater burden. Results of multiple regression models showed that positive affect (β = -.24, p = .03) and negative affect (β = .35, p = .01) were significant predictors of burden even while controlling for other constructs including types of coping, compassion towards other, and assessment of family dynamics. Understanding the relationship between these constructs will allow for more individualized interventions to be created to help reduce the level of burden that dementia caregivers experience.

